# Associations of modifiable and non-modifiable risk factors with cognitive functions – a prospective, population-based, 17 years follow-up study of 3,229 individuals

**DOI:** 10.1186/s13195-024-01497-6

**Published:** 2024-06-26

**Authors:** Isabelle Glans, Katarina Nägga, Anna-Märta Gustavsson, Erik Stomrud, Peter M Nilsson, Olle Melander, Oskar Hansson, Sebastian Palmqvist

**Affiliations:** 1 Clinical Memory Research Unit, Department of Clinical Sciences, Lund University, Malmö, Sweden; 2 Memory Clinic, Skåne University Hospital, Malmö, Sweden; 3 Department of Acute Internal Medicine and Geriatrics, Linköping University, Linköping, Sweden; 4 Department of Clinical Science, Lund University, Skåne University Hospital, Malmö, Malmö, Sweden

## Abstract

**Background:**

Although several cardiovascular, demographic, genetic and lifestyle factors have been associated with cognitive function, little is known about what type of cognitive impairment they are associated with. The aim was to examine the associations between different risk factors and future memory and attention/executive functions, and their interaction with *APOE* genotype.

**Methods:**

Participants from a large, prospective, population-based, Swedish study were included (*n* = 3,229). Linear regression models were used to examine baseline hypertension, body mass index (BMI), long-term glucose levels (HbA_1c_), different lipid levels, physical activity, alcohol consumption, smoking, education, *APOE* genotype, age and sex. All models were adjusted for follow-up time and basic demographics, and, in a second step, all significant predictors were included to examine independent effects. Follow-up outcomes were memory and attention/executive functions.

**Results:**

The mean age at baseline was 56.1 (SD 5.7) years and 59.7% were women. The mean follow-up time was 17.4 (range 14.3–20.8) years. When examining independent effects, *APOE* ε4 genotype(*p* < 0.01), and higher HbA_1c_(*p* < 0.001), were associated with future low memory function. Higher BMI (*p* < 0.05), and HbA_1c_(*p* < 0.05), lower high-density lipoprotein cholesterol (HDL-C)(*p* < 0.05)and stroke(*p* < 0.001) were associated with future low attention/executive function. The strongest factors associated with both better memory and attention/executive functions were higher education and alcohol consumption. Further, significant interaction effects between predictors and *APOE* genotype were found. For memory function, the protective effects of education were greater among ɛ4-carriers(*p* < 0.05). For attention/executive function, the protective effects of alcohol were greater among ɛ2 or ɛ4-carriers(*p* < 0.05). Also, attention/executive function was lower among ɛ4-carriers with higher BMI(*p* < 0.05) and ɛ2-carriers with higher HbA1c-levels(*p* < 0.05).

**Conclusions:**

Targeting cardiovascular risk factors in mid-life could have greater effect on future attention/executive functions rather than memory, whereas targeting diabetes could be beneficial for multiple cognitive domains. In addition, effects of different risk factors may vary depending on the *APOE* genotype. The varied cognitive profiles suggest that different mechanisms and brain regions are affected by the individual risk factors. Having detailed knowledge about the specific cognitive effects of different risk factors might be beneficial in preventive health counseling.

**Supplementary Information:**

The online version contains supplementary material available at 10.1186/s13195-024-01497-6.

## Introduction

Cognitive decline increases with age but is not an inevitable consequence of healthy aging. Understanding underlying mechanisms could be of great importance for targeting modifiable risk factors to delay or prevent the onset of cognitive impairment. Total cardiovascular risk has previously been associated with increased cognitive decline [1]. Specific co-morbidities, e.g. diabetes, have been shown to be a modifiable risk factor for cognitive impairment [2, 3], whereas targeting hypertension has presented conflicting results. Even though there are some well-established risk factors for dementia such as diabetes, hypertension, obesity, low education and harmful use of alcohol [4–6], large longitudinal studies on how various types of risk factors contribute to cognitive decline in different cognitive domains are lacking. A deeper understanding of which cognitive functions are affected by different risk factors could provide useful information about potential underlying mechanism of the cognitive impairment and more precise preventive health counceling about the dangers of certain conditions. The use of short follow-up periods and the absence of adjustment for a range of potential confounding factors in the previous studies also contribute to uncertain conclusions regarding the impact of various risk factors [7]. 

The aim of this study was to investigate how different risk factors contribute to future memory and attention/executive function, respectively. Furthermore, we examined how they interacted with *APOE* genotype, as *APOE* ɛ4 is a well-established risk factor/marker for Alzheimer’s disease (AD) [8] and *APOE* ɛ2 have been shown both being a risk factor for cardiovascular disease [9] but in a meta-analysis being a protective factor for myocardial infarction [10]. This was carried out in a large population-based study (*n* = 3,229) including extensively characterized participants with a follow-up of 14–21 years.

## Methods

### Study population

Data was obtained from the population-based prospective Malmö Diet and Cancer Study (MDCS). Between 1991 and 1996, men born 1923–1945 and women born 1923–1950 who were living in Malmö were invited to participate. Baseline examination included body composition measurements, blood pressure, baseline questionnaire and dietary assessments. In the self-administered baseline questionnaire (http://links.lww.com/WNL/A48) education, occupation, current medication and family history of diseases in close relatives were assessed. Dietary assessments included: (1) a prospective seven-day diet record, (2) a self-administered food frequency questionnaire and (3) a 45–60 min interview with trained personnel. Exclusion criteria for participating in Malmö Diet and Cancer Study (MDCS) included language difficulties and intellectual disability, which could preclude participants from filling in questionnaires properly. From MDCS, a randomly selected population were invited to further baseline examinations with fasting plasma samples and carotid artery examination, forming the cardiovascular cohort (MDSC-CV). In 2007–2012 participants of MDSC-CV were invited to a re-examination where 76% of the surviving population participated [11]. At follow-up, cognitive assessments were performed, including the Mini-Mental State Examination (MMSE) [12] and A Quick Test of cognitive speed (AQT) [13].

All participants received information about the study and gave written consent to participate. Ethical approval was given by the Ethical Committee of Lund University, Lund, Sweden **(**LU 51–90, LU 532–2006).

### Demographic predictors of cognitive function

Sociodemographic factors included age, sex and level of education. Based on information from the baseline self-reported questionnaire, variables were divided as follows: Education level into three levels as per study design: primary/elementary school (≤ 8 years), secondary school/high school (9–12 years), or higher education/university (≥ 13 years); Smoking status into smokers, former smokers, and never smokers; and physical activity as metabolic equivalent hours/week (METh/week) where one METh is defined as the metabolic intensity when a person is at rest. METh/week was computed by multiplying time (hours) spent on each activity by the respective metabolic equivalent task (intensity) factor (MET) [14]. Information on alcohol consumption (g/day) was derived both from the food frequency questionnaire and the seven-day diet record. Alcohol consumption was divided into quartiles, and 14 g of pure ethanol was considered to represent one standard drink, from the National Institution on Alcohol Abuse and Alcoholism [15]. Zero-consumers had reported no consumption during the past year. In sensitivity analyses, other stratifications of alcohol consumption were used. Triglycerides (mmol/l), cholesterol (mmol/l), high-density lipoprotein cholesterol (HDL-C) (mmol/l), and low-density lipoprotein cholesterol (LDL-C) (mmol/l) and blood glucose were measured in serum after an overnight fast at the baseline visit, using standard procedures at the Department of Clinical Chemistry, Malmö, Sweden [16]. Systolic blood pressure was measured after 10 min of rest in a horizontal position. A diagnosis of hypertension was derived from the baseline questionnaire. We calculated body mass index (BMI) as kg/m^2^. *APOE* genotype analyzed from blood was divided into four groups: ɛ2-carrier (ɛ2/ɛ2, ɛ2/ɛ3), ɛ3/ɛ3, ɛ4-carrier (ɛ3/ɛ4, ɛ4/ɛ4) and ɛ2/ɛ4, with ɛ3/ɛ3 as reference group in the statistical models, since ɛ4 is associated with increased risk of AD [8] and ɛ2 with increased cardiovascular disease (but lower risk of AD) [17]. History of stroke was derived from the questionnaire (prevalent at baseline) or during follow-up from the Swedish National Patient Register (NPR). That is, the variable “stroke” denoted either prevalent or incident stroke. The NPR covers both the Swedish Inpatient Register and the hospital-based outpatient register.

### Cognitive outcomes

The primary outcomes were memory and attention/executive functions. Memory function was measured using the *delayed word recall* part (remembering 0–3 words previously presented) and *orientation to time and location* (0–10 points for remembering e.g. present year, month, day of the week, and what floor the examination is taking place on, name of the building etc.). These two parts of the MMSE (examining both episodic and semantic memory functions) have previously extensively been used for assessing the memory impairment seen in AD, e.g. for identifying early AD-related memory impairment in non-demented individuals [18–20] and for differential diagnosis of the memory impairment seen in AD versus the cognitive impairment seen in dementia with Lewy bodies [21].

*Attention and executive functions* were examined using A Quick Test of cognitive speed (AQT), which has a high sensitivity for impaired attention (processing speed) and executive function (set-shifting) [13]. The AQT score constitutes the number of seconds it takes to fulfil each test plate of colours and shapes of figures, thus higher score equals worse performance. Part three (naming first colour, then shape) of the AQT is the most extensively validated part as a sensitive measure of attention/executive function and was used as outcome [13, 22–24]. Both the memory score and the attention/executive score were converted to z-scores, based on the distribution in the present population, for easier comparison of estimates. The attention/executive score was also inverted so that higher scores equal better performance, for both cognitive domains.

### Statistical analysis

Chi-square test (for binary/categorical) variables and the Mann-Whitney U test (for continuous variables) were used for group comparisons. Continuous data were converted to z-scores based on the distribution in the present population for easier comparison of estimates. Multivariate linear regression models were used to examine associations between non-modifiable/modifiable risk factors (i.e., predictors) and subsequent cognitive performance (either memory or attention/executive z-scores). Associations between the predictor and future cognitive performance were examined in three steps: (1) univariate models including just the predictor and time from baseline to cognitive testing at follow-up; (2) basic models, including the predictor, age, sex, education, prevalent (at baseline) or incident (during follow-up) stroke (from now on referred collectively as “stroke”), and time from baseline to cognitive testing at follow-up; and (3) multivariable models, including the predictors that were significant in the basic models, and the same adjusting co-variates as in the basic models. To avoid collinearity issues, HbA_1c_, diabetes, and plasma glucose levels were not entered in the same model (only HbA_1c_ in the multivariable model). Logistic regression models were used to examine predictors of drop-out from the study (i.e., baseline data available but no follow-up examination), adjusted for age, sex, education, time to follow-up and stroke.

An *APOE* interaction analysis was performed to examine potential interactions between risk factor and *APOE* genotype. *APOE* genotype was divided into four groups: ɛ3/ɛ3 (reference), ɛ2/ɛ2 or ɛ2/ɛ3, ɛ3/ɛ4 or ɛ4/ɛ4, and ɛ2/ɛ4.

All statistical analyses were performed using R version 4.2.1 (R Foundation for Statistical Computing). A *p*-value < 0.05 was considered to indicate statistical significance.

## Results

### Baseline characteristics

Participation in the MDCS reached 40.8% of the eligible population [25]. In total 30,446 attended in at least a part of the baseline examination.

**Fig. 1:**
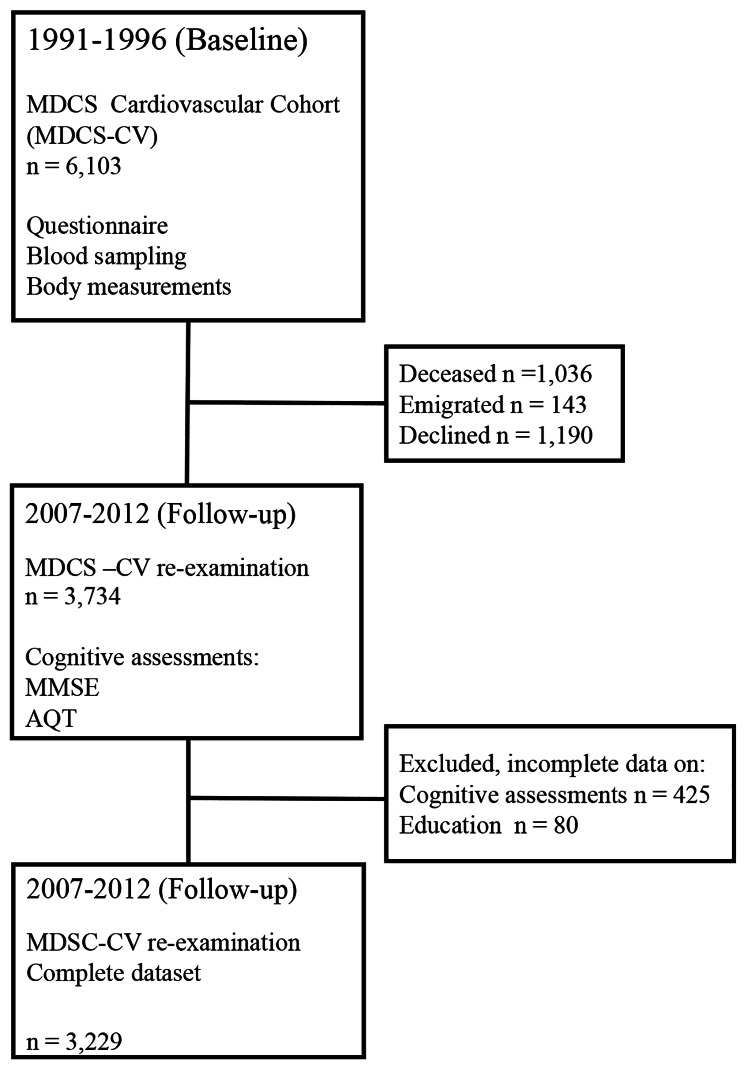
Flow chart describing the Malmö Diet and Cancer Study (MDCS) Cardiovascular Cohort at baseline 1991–1994 and follow-up 2007–2012

In 2007–2012 participants of MDSC-CV were invited to a re-examination where 3,734 people participated. Participants declining follow-up had generally poorer health status than the included participants. Reasons for non-participation included unwillingness, sickness, deceased or lack of contact information in registers. Individuals participating at follow-up in the MDSC-CV (2007–2012) were included in the study (*n* = 3,734). Reasons for non-participation at follow-up are shown in Fig. 1. Participants with incomplete data on cognitive assessments (*n* = 425) and education (*n* = 80) were excluded, resulting in a complete dataset of 3,229 individuals, which was used for the main analysis. A flow-chart of the inclusion is shown in Fig. 1. Table 1 presents characteristics of the study population. The mean age was 56.1 (SD 5.65, range 45.8–68.0) years at baseline and 59.7% were women. Mean time to the follow-up visit was 17.4 (SD 1.43, range 14.3–20.8) years [11]. 

**Table 1:** Characteristics of the Study population

	Total (*n* = 3,229)
**Age, y**	56.1 (5.65, 45.8–68.0)
**Education level**, ***n*(%)**	
< 8 years	1304 (40.4)
9–12 years	1212 (37.5)
≥ 13 years	713 (22.1)
**Time to follow-up visit, y**	17.4 (1.43, 14.3–20.8)
**Follow-up AQT**	
Part 3 (color and form, seconds)	74.0 (20.2, 35–295)
**Follow-up MMSE**	
Total score	28.1 (1.81, 11–30)
Orientation and memory scores	12.1 (1.02, 4–13)
**Hypertension**	
Hypertension, from baseline questionnaire, n (%)	444 (13.8)
Systolic blood pressure (mmHg)	139 (18.0)
**Lipid levels**	
Total cholesterol (mmol/l)	6.13 (1.07)
HDL-C (mmol/l)	1.41 (0.372)
LDL-C (mmol/l)	4.13 (0.976)
Triglycerides (mmol/l)	1.29 (0.692)
**Lipid lowering medication, n (%)**	63 (2.0)
**Diabetes**	
Diabetes at baseline, n (%)	179 (5.5)
HbA_1c_ (%)	4.80 (0.598)
Fasting glucose (mmol/l)	5.01 (0.997)
**Stroke (prevalent or incident), n (%)**	241 (7.5)
**Body Mass Index** ^**a**^	25.4 (3.69)
**Smoking**, ***n*(%)**	
Current smoker	723 (22.4)
Former smoker	1137 (35.2)
Never smoker	1368 (42.4)
**Alcohol consumption, g/day**	10.8 (11.6)
Q1 (≤ 0.23 standard drinks/day)	0.61 (0.77)
Q2 (0.24–0.56 standard drinks/day)	5.15 (1.60)
Q3 (0.57–1.01 standard drinks/day)	11.01 (2.09)
Q4 (≥ 1.02 standard drinks/day)	26.33 (12.41)
***APOE*** **genotype, n (%)**	
ε2/ε2 or ε2/ε3	397 (12.3)
ε3/ε3	1780 (55.1)
ε2/ε4	79 (2.4)
ε3/ε4 or ε4/ε4	812 (25.1)
**Physical activity (METh/week)** ^**b**^	3.41 (1.15)

Data are shown as mean (SD, range) if not otherwise specified. All data represent baseline data except for MMSE and AQT scores at follow-up. Stroke is accounted for as prevalent at baseline or incident during follow-up

^a^ Calculated as weight in kilograms divided by height in meters squared

^b^ Physical activity as metabolic equivalent hours/week (METh/week). One METh is defined as the metabolic intensity when a person is at rest. METh/week was computed by multiplying time (hours) spent on each activity by the respective metabolic equivalent task (intensity) factor (MET).

### Association between risk factors and future memory function

**Fig. 2:**
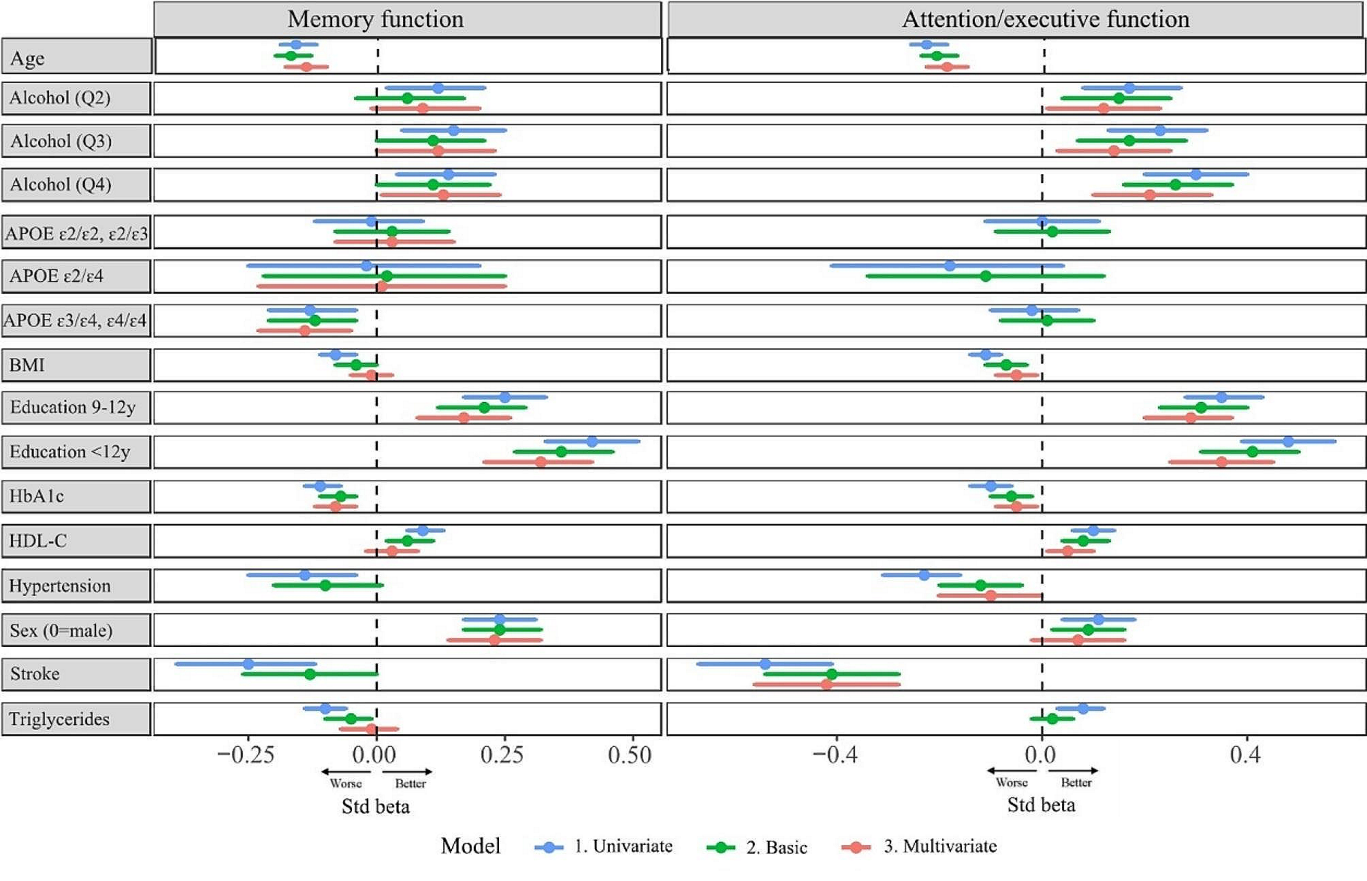
Predictors of future memory and attention/executive function. All continuous variables used as z-scores. Alcohol consumption in quartiles with lowest quartile as reference: Q1 ≤ 0.23 standard drinks/day (≤ 3.37 g/day); Q2 0.24–0.56 standard drinks/day (3.38–7.83 g/day); Q3 0.57–1.01 standard drinks/day (7.84–15.2 g/day); Q4 ≥ 1.02 standard drinks/day, (≥ 15.3 g/day). Univariate model: Including only the predictor and time between baseline and follow-up. Basic model: Including the predictor, adjusted for age, sex, education, time between baseline and follow-up and prevalent or incident stroke. Multivariable model: One model combining all significant predictors from the basic models, adjusted for age, sex, education, time between baseline and follow-up, prevalent or incident stroke and blood lipid lowering medication

Associations between modifiable and non-modifiable risk factors in relation to future memory function are presented in Table 2; Fig. 2. The multivariable models examined the independent effects of the predictors on cognitive performance measured as z-scores (i.e., SD). They showed that the strongest protective predictor was education, where both 9–12 years (β = 0.17, 95%CI:0.08–0.26) and > 12 years of education (β = 0.32, 95%CI:0.21–0.42) were associated with better memory function (using ≤ 8 years as reference). The second strongest protective predictor was alcohol consumption, where consumption in both the second highest quartile, Q3 0.6-1.0 standard drinks/day, (β = 0.12, 95%CI:0.00-0.23) and highest quartile, Q4 ≥ 1.1 standard drinks/day, (β = 0.13, 95%CI:0.01–0.24) were associated with better memory function using the lowest consumption quartile, Q1 < 0,2 standard drinks/day, as reference. The robustness of this association was further examined in sensitivity analyses (end of Results section). Female sex was associated with better memory performance (β = 0.23, 95%CI:0.14–0.32). Presence of *APOE* ɛ4/ɛ4 or ɛ3/ɛ4 genotype (β=-0.14, 95%CI:-0.23– -0.05), age (β=-0.14, 95%CI:-0.18– -0.10), and higher HbA_1c_ (β=-0.08, 95%CI:-0.12– -0.04), were all independently associated with lower memory function. Non-significant predictors are shown in eTable 1 and included for example a diagnosis of hypertension at baseline, physical activity and former/current smoking.

**Table 2:** Significant predictors of future memory function

Baseline predictor ^a^	Univariate models ^b^	Basic models ^c^	Multivariable model ^d^
	β (95% CI)	β (95% CI)	β (95% CI)
**Age**	-0.16 (-0.19 – -0.12)***	-0.17 (-0.20 – -0.13)***	**-0.14** (-0.18 – -0.10)***
**Alcohol consumption** ^**e**^			
Q1 (≤ 0.23 standard drinks/day) Q2 (0.24–0.56 standard drinks/day) **Q3 (0.57–1.01 standard drinks/day)** **Q4 (≥ 1.02 standard drinks/day)**	Reference 0.12 (0.02–0.21)* 0.15 (0.05–0.25)** 0.14 (0.04–0.23)**	Reference 0.06 (-0.04–0.17) 0.11 (0.00–0.21)* 0.11 (-0.00–0.22)	Reference 0.10 (-0.01–0.20) 0.12 (0.00– 0.23)* 0.13 (0.01–0.24)*
***APOE genotype***			
ɛ3/ɛ3 ɛ2/ɛ2 or ɛ2/ ɛ3 ɛ2/ɛ4 ɛ3/ɛ4 or ɛ4/ɛ4	Reference -0.01 (-0.12–0.09) -0.02 (-0.25–0.20) -0.13 (-0.21 – -0.04)**	Reference 0.03 (-0.08–0.14) 0.02 (-0.22 – 0.25) -0.12 (-0.21 – -0.04)**	Reference 0.03 (-0.08–0.15) 0.01 (-0.23–0.25) -0.14 (-0.23 – -0.05)**
BMI	-0.08 (-0.11 – -0.04)***	-0.04 (-0.08 – -0.00)*	-0.01 (-0.05–0.03)
Diabetes ^f^	-0.36 (-0.51 – -0.21)***	-0.29 (-0.44 – -0.13)***	Not included ^g^
**Education**			
≤8 years **9–12 years** **>12 years**	Reference 0.25 (0.17–0.33)*** 0.42 (0.33–0.51)***	Reference 0.21 (0.12–0.29)*** 0.36 (0.27–0.46)***	Reference 0.17 (0.08–0.26)*** 0.32 (0.21–0.42)***
Glucose (plasma)	-0.10 (-0.14 – -0.07)***	-0.09 (-0.12 – -0.05)***	Not included ^g^
**HbA**_**1c**_	-0.11 (-0.14 – -0.07)***	-0.07 (-0.11 – -0.04)***	-0.08 (-0.12 – -0.04)***
HDL-C	0.09 (0.06–0.13)***	0.06 (0.02–0.11)**	0.03 (-0.02–0.08)
**Sex (0 = men, 1 = women)**	0.24 (0.17–0.31)***	0.24 (0.17 − 0.32) ***	0.23 (0.14–0.32)***
Systolic blood pressure	-0.11 (-0.14 – -0.07)***	-0.04 (-0.08 – -0.00)*	-0.03 (-0.07–0.01)
Triglycerides	-0.10 (-0.14 – -0.06)***	-0.05 (-0.10 – -0.01)*	-0.01 (-0.07–0.04)

Only predictors that were significant in the basic model are shown in Table 2. The predictors that were non-significant in the basic model are shown in eTable 1 and included: Carotid stenosis, hypertension, LDL-C, physical activity, prevalent or incident stroke and smoking. Predictors in bold represent the significant predictors from the multivariable model

^a^ All continuous variables used as z-scores (i.e., not alcohol, *APOE*, diabetes, education, hypertension and sex)

^b^ Including only the predictor and time between baseline and follow-up

^c^ Including the predictor adjusted for age, sex, education, time between baseline and follow-up, and prevalent or incident stroke

^d^ One model combining all significant predictors from the basic models, adjusted for age, sex, education, time between baseline and follow-up, prevalent or incident stroke and blood lipid lowering medication

^e^ Alcohol consumption in quartiles with lowest quartile as reference: Q1 ≤ 0.23 standard drinks/day (0–3.37 g/day); Q2 0.24–0.56 standard drinks/day (3.38–7.83 g/day); Q3 0.57–1.01 standard drinks/day (7.84–15.2 g/day); Q4 ≥ 1.02 standard drinks/day, (≥ 15.3 g/day)

^f^ Defined as a diabetes diagnosis entered in the baseline questionnaire or having fasting plasma glucose levels > 6mmol/L at baseline

^g^ To avoid collinearity issues, HbA_1c_, diabetes, and plasma glucose levels were not entered in the same model (only HbA_1c_ in the multivariable model)

* *p* < 0.05, ** *p* < 0.01, *** *p* < 0.001

### Association between risk factors and future attention/executive function

Associations between modifiable and non-modifiable risk factors and future attention/executive function are presented in Table 3; Fig. 2. The multivariable models examined repeatedly the independent effects of the predictors on cognitive performance measured as z-scores (i.e., SD). From the multivariable models, education was again shown to be the strongest protective predictor, where both 9–12 years (β = 0.29, 95%CI:0.20–0.37) and > 12 years of education (β = 0.35, 95%CI:0.24–0.45) were associated with better attention/executive function (using ≤ 8 years as reference). The second strongest protective factor was alcohol, where consumption in the upper three quartiles, Q2 0.2–0.5 standard drinks/day, (β = 0.12, 95%CI:0.02–0.23), Q3 0.6-1.0 standard drinks/day, (β = 0.14, 95%CI0.04-0.25) and highest Q4 ≥ 1.1 standard drinks/day, (β = 0.21, 95%CI:0.11–0.33) all were associated with a better attention/executive function using the lowest consumption quartile, Q1 < 0,2 standard drinks/day, as reference. The robustness of this association was further examined in sensitivity analyses (end of Results section). Furthermore, higher HDL-C was associated with better attention/executive performance (β = 0.06, 95%CI:0.01–0.10). Presence of stroke (β=-0.41, 95%CI:-0.56– -0.27), age (β= -0.19, 95%CI:-0.23– -0.15), higher BMI (β=-0.04, 95%CI:-0.09– -0.00), and higher HbA_1c_ (β=-0.05, 95%CI:-0.09– -0.01) were all independently associated with lower attention/executive function. Non-significant predictors are shown in eTable 2 and included for example *APOE*-genotype, physical activity, and smoking. See also sensitivity analyses at the end of the Results section.

**Table 3:** Significant predictors of future attention/executive function

Baseline predictor ^a^	Univariate model ^b^	Basic model ^c^	Multivariable model ^d^
	β (95% CI)	β (95% CI)	β (95% CI)
**Age**	-0.23 (-0.26 – -0.19)***	-0.20 (-0.24 – -0.17)***	-0.19 (-0.23 – -0.15)***
**Alcohol consumption** ^**e**^			
Q1 (**≤** 0.23 standard drinks/day) **Q2 (0.24–0.56 standard drinks /day)** **Q3 (0.57–1.01 standard drinks/day)** **Q4 (≥ 1.02 standard drinks/day)**	Reference 0.17 (0.08–0.27)*** 0.23 (0.13–0.32)*** 0.30 (0.20–0.40)***	Reference 0.15 (0.04–0.25)** 0.17 (0.07–0.28)*** 0.26 (0.16–0.37)***	Reference 0.12 (0.02–0.23)* 0.14 (0.04–0.25)** 0.21 (0.11–0.33)***
**BMI**	-0.11 (-0.14 – -0.08)***	-0.07 (-0.11 – -0.03)***	-0.04 (-0.09 – -0.00)*
Diabetes ^f^	-0.44 (-0.59 – -0.29)***	-0.37 (-0.52 – -0.21)***	Not included ^g^
**Education**			
≤8 years **9–12 years** **>12 years**	Reference 0.35 (0.28–0.43)*** 0.48 (0.39–0.57)***	Reference 0.31 (0.23–0.40)*** 0.41 (0.31–0.50)***	Reference 0.29 (0.20–0.37)*** 0.35 (0.24–0.45)***
Glucose (plasma)	-0.10 (-0.13 – -0.06) ***	-0.07 (-0.10 – -0.03)***	Not included ^g^
**HbA**_**1c**_	-0.10 (-0.14 – -0.06)***	-0.06 (-0.10 – -0.02)***	-0.05 (-0.09 – -0.01)*
**HDL-C**	0.10 (0.06–0.14)***	0.08 (0.04–0.13)***	0.06 (0.01–0.10)*
Hypertension ^h^	-0.26 (-0.36 – -0.16)***	-0.14 (-0.24 – -0.03)*	-0.10 (-0.21–0.01)
Sex (0 = men, 1 = women)	0.11 (0.04–0.18)**	0.09 (0.02–0.16)*	0.07 (-0.02–0.16)
Systolic blood pressure	-0.12 (-0.15 – -0.08)***	-0.03 (-0.07–0.01)	Not included
**Stroke (prevalent or incident)**	-0.54 (-0.67 – -0.41)***	-0.41 (-0.54 – -0.28)***	-0.41 (-0.56 – -0.27)***

Only predictors that were significant in the basic model are shown in Table 3. The predictors that were non-significant in the basic model are shown in eTable 2 and included: *APOE*, carotid stenosis, LDL-C, physical activity, smoking, systolic blood pressure and triglycerides. Predictors in bold represent the significant predictors from the multivariable model

^a^ All continuous variables are shown as z-scores, (i.e., not alcohol, diabetes, education and sex)

^b^ Including only the predictor and time between baseline and follow-up

^c^ Including the predictor adjusted for age, sex, education, time between baseline and follow-up and prevalent or incident stroke

^d^ One model combining all significant predictors from the basic models, adjusted for age, sex, education, time between baseline, prevalent or incident stroke and follow-up and blood lipid lowering medication

^e^ Alcohol consumption in quartiles with lowest quartile as reference: Q1 ≤ 0.23 standard drinks/day (0–3.37 g/day); Q2 0.24–0.56 standard drinks/day (3.38–7.83 g/day); Q3 0.57–1.01 standard drinks/day (7.84–15.2 g/day); Q4 ≥ 1.02 standard drinks/day, (≥ 15.3 g/day)

^f^ Defined as a diabetes diagnosis entered in the baseline questionnaire or having fasting plasma glucose levels > 6mmol/L at baseline

^g^ To avoid collinearity issues, HbA_1c_, diabetes, and plasma glucose levels were not entered in the same model (only HbA_1c_ in the multivariable model)

^h^ Defined as hypertension from baseline questionnaire

* *p* < 0.05, ** *p* < 0.01, *** *p* < 0.001

### Interaction effects between APOE-genotype and future memory function

Interactions between significant predictors (from the multivariable models) and *APOE* genotype on future memory function are presented in eTable 3 and eFigure 1. The protective effect of education was significantly lower among *APOE* ɛ4/ɛ4 and ɛ3/ɛ4-carriers and those with > 12 years of education (interaction *p-value < 0.05*). Further, individuals with *APOE* ɛ2/ɛ4 and higher HDL-C had better memory function (*p* < 0.05). *APOE* ɛ3/ɛ4 and ɛ4/ɛ4-carriers with stroke had lower memory performance (*p* < 0.05).

### Interaction effects between APOE-genotype and future attention/executive function

The protective effects of alcohol on attention/executive function were highest among ɛ2-carriers (ɛ2/ɛ3) and ɛ4-carriers (ɛ3/ɛ4 and ɛ4/ɛ4), (interaction *p-values < 0.05*). Individuals with *APOE* ɛ4/ɛ4 and ɛ3/ɛ4 with higher BMI had lower attention/executive function, (*p* < 0.05). ɛ2-carriers (ɛ2/ɛ2, ɛ2/ɛ3 or ɛ2/ɛ4) with increasing HbA_1c_-levels had lower attention/executive function (interaction *p-value < 0.05*). For memory function, *APOE* ɛ4/ɛ4 and ɛ3/ɛ4-carriers with stroke performed significantly lower on attention/executive function (*p* < 0.05). (eTable 3 and eFigure 2)

### Sensitivity analyses of alcohol, lipids, stroke, hypertension and predictors of attending the follow-up visit

Using the original grouping of alcohol consumption in quartiles, participants reporting zero alcohol consumption were excluded (*n* = 400), since this group is known to introduce bias related to previous alcoholism or other co-morbidities causing the individual to stop drinking alcohol [26]. In this population of alcohol consumers (*n* = 2829), higher alcohol consumption, adjusted for age, sex, education and time to follow-up, was still associated with better attention/executive function for Q2 (*p* < 0.05), Q3 (*p* < 0.01) and Q4 (*p* < 0.001). All quartiles but the lowest (reference) were also significant in the multivariable step (*p* < 0.05 − 0.01). Alcohol consumption was however no longer a significant predictor of better memory function, when excluding zero-consumers (eTable 4). Finally, the population was stratified based on the cut-off of 168 g/week (1.7 standard drinks/day) from the Lancet Commission [5] (suggested to be associated with increased risk of dementia), to examine a potential U-shaped effect whereby the very highest consumption would have a negative effect on cognition. In this “risk consumption” group (*n* = 343) we did not, however, find a lower performance in any cognitive function, compared with those with consumption below the cut-off, even when excluding zero consumers (eTable 4).

To examine if the seemingly positive effect of alcohol consumption was caused by a “survival bias”, alcohol consumption was examined as a predictor of drop-out during study follow-up (adjusted for age, sex, education, and stroke). This showed the opposite relationship, i.e., that higher alcohol consumption was associated with higher likelihood of turning up at the follow-up visit (< 0.001 for quartiles 2–4; eTable 5). Participants with higher education were also more likely to participate at follow-up. We found, however, that higher HbA_1c_, a diagnosis of hypertension at baseline, higher age, and stroke were associated with lower likelihood of attending the follow-up visit (eTable 5). No associations were found for APOE genotype, BMI and cholesterol-levels, and attending the follow-up visit.

To examine a potential U-shaped association of low-density lipoprotein cholesterol (LDL-C) and attention/executive function, sensitivity analysis with LDL-C-levels in quartiles was carried out. We found no significant association with attention/executive function with LDL levels and quartile one, three or four using quartile two as reference (data not shown).

Since stroke is a known risk factor for cognitive impairment, we also performed a sensitivity analysis excluding those with prevalent stroke at baseline. In the multivariable analysis for memory function, the significant predictors remained the same except for alcohol consumption which was no longer significant at any level of consumption. In the multivariable analysis for attention/executive function, the same variables were significant as in the main result (data not shown).

When adjusting the basic model for antihypertensive treatment, hypertension was still a significant predictor of attenttion/executive function, but not memory (data not shown), and it did not change any of the significant predictors in the multivariable models.

## Discussion

In this longitudinal, population-based prospective cohort study of 3,229 individuals with 17 years of follow-up, we found that cardiovascular risk factors such as higher BMI, lower HDL-C and stroke were associated with lower attention/executive function, while having an *APOE* ɛ4-allele was associated with poorer memory function. Higher HbA_1c_ was associated with lower performance in both cognitive domains. Higher education and higher alcohol consumption were associated with both better memory and attention/executive functions. Interaction effects were found between predictors and *APOE* genotype. For memory function, the protective effects of education were greater among ɛ4-carriers. For attention/executive function, the protective effects of alcohol were largest among ɛ2 or ɛ4-carriers. Also, attention/executive function were lower among ɛ4-carriers with higher BMI and ɛ2-carriers with higher HbA1c-levels.

The main strengths of this study were the prospective study design, large sample size, long follow-up time, and the analysis of a wide range of risk factors in multivariable models to examine independent effects. In a previous cross-sectional large population-based study, no association between *APOE* genotype and cognition were found, concluding the need for further longitudinal studies [27]. Here we show a significant relationship between *APOE* ɛ4 and future low memory function. The most likely mechanism for this association is the well-known association with β-amyloid accumulation, which in turn facilitates tau accumulation (the hallmark pathologies of Alzheimer’s disease) that starts around the entorhinal cortex and hippocampus (key regions for the memory function) [28, 29]. 

Several cardiovascular risk factors are known to be associated with cognitive impairment [30–32], but here we show that they are specifically associated with future attention/executive function (Table 3; Fig. 2). These risk factors included higher BMI, elevated HbA_1c_ and low HDL-C. Hypertension has previously been associated with cognitive impairment in numerous studies [33–35], whereas others have not been able to replicate that [3, 36]. In this present study, we found an association, specifically with attention/executive function both in univariate analyses and when adjusting for age, gender, education and stroke. The association did, however, not remain significant in the multivariable model, suggesting that the effect to some extent could be mediated by any of the other risk factors, or that it co-varies with another stronger risk factor such as stroke. The association of cardiovascular risk factors with specifically attention/executive function, and not memory, suggest that there is another pathophysiological mechanism behind this type of cognitive decline. One possible explanation is that these risk factors are more related to arteriolosclerosis (arterial stiffness) and venous collagenosis [37] in the brain, which could contribute to attention/executive impairment. This suggests targeting cardiovascular risk factors in mid-life could have greater impact on future attention/executive function, but less so on memory function. High HbA_1c_ levels were, however, associated with lower function in both cognitive domains, and consistent results were found with increasing plasma glucose and prevalence of diabetes. This indicates that high glucose levels are associated with lower cognitive functions in multiple cognitive domains, through potentially different mechanisms.

Higher education was, as expected, associated with better cognitive performance in both domains, as low education is a well-known risk factor for cognitive decline and dementia. In the present study, higher alcohol consumption was associated to both better memory and attention/executive function. After excluding zero-consumers this association was, however, only significant for attention/executive function, making this association more robust than that for memory. The sensitivity analysis suggests that the seemingly positive effect of alcohol on memory performance is not caused by increasing alcohol consumption, but instead by worse memory performance in the zero-consumer group (potentially related to co-morbidities and previous alcoholism). Similar positive effects of alcohol on cognitive function have previously been shown [38, 39]. The potential mechanism behind alcohol consumption and better cognitive performance may be related to potentially better cardiovascular health [40] even though this has not been confirmed in a recent study using Mendelian randomization [41]. The suggested beneficial cardiovascular effect has been indicated to be U-shaped, whereby both very low and very high consumption have a negative effect [42]. We could, however, not find that high consumption (as defined by the Lancet Commission [5]) was associated with worse cognitive function (eTable 4). Nonetheless, it is important to note that this study only examined the effect of alcohol on cognitive function and study adherence, but not other known negative effects of alcohol [43, 44]. Although we see a robust positive finding between alcohol consumption and attention/executive function, we do not make any claims that alcohol is overall beneficial or recommend increased alcohol consumption in low consumers. Further, alcohol consumption was self-reported which can introduce biases and it may also reflect other factors influencing cognition such as socioeconomic status, which was only indirectly adjusted for using for example education level [45]. Cultural or religious differences could possibly also affect the results. 

An interaction effect between *APOE* genotype and diabetes on cognitive function and dementia has previously been shown, though primarily between *APOE* ɛ4 and diabetes [46, 47]. In this study, we instead found that *APOE* ɛ2-carriers with increased HbA_1c_ had greater impairment of attention/executive function. *APOE* ɛ2 is a known mediator of hyperlipidaemia, because of its inaccurate binding to LDL receptors, and can in the presence of other environmental factors increase the risk of atherosclerosis [9], which could be an explanation of the observed interaction effect in that this provides a synergistic detrimental effect with HbA_1c_ on the vascular system with a downstream effect on attention/executive function. Hyperglycaemia is known to be associated to arterial stiffness with negative effects on attention/executive function [48, 49]. *APOE* ɛ4-carriers with stroke had lower cognitive function in both cognitive domains, which is in agreement with the previously found synergistic effect of having both AD pathology (accelerated by ɛ4-carriership) and cerebrovascular disease [50–53]. 

In this study, we did not find any effects of physical activity on cognitive function. This is in line with a recent large review and meta-analysis [54], concluding that positive findings in previous studies may be due to low statistical power, publication bias, and unseemly adjustment for baseline differences.

This study has some limitations. As in the case of all non-randomized, observational studies, other confounding factors might be present, although we have adjusted for multiple potential confounding and mediating factors at baseline. Further, a potential survival bias could have been introduced at follow-up since hypertension and higher HbA_1c_ levels were predictors of drop-out during follow-up (eTable 5) potentially minimizing their negative effect on cognition. They were, however, still significant predictors of cognition, so this potential bias would only have affected the effect size. Another limitation is the cognitive tests, which only capture some aspects of the cognitive domains memory and attentions/executive function. The lack of baseline cognitive assessments is also a limitation. The findings should be validated with more extensive neuropsychological tests. For alcohol consumption, a positive association of higher consumption and cognitive function can potentially be biased if only selected, more healthy, higher consumers attend follow-up due to a large drop-out related to the known negative health effects of high alcohol consumption [43, 44]. However, in our drop-out analysis we found the opposite, i.e., that higher alcohol consumption at baseline predicted higher probability of attending the follow-up visit (eTable 5). We can therefore exclude that such a survival bias caused the somewhat controversial finding of a positive effect of higher alcohol consumption on better cognitive function.

## Conclusion

In this prospective study of 3,229 middle-aged individuals with 17-years of follow-up, *APOE* ε4 genotype was associated with future lower memory function. High BMI, low HDL-C and stroke were associated with future lower attention/executive functions, but not future memory function. Diabetes was associated with lower cognitive function in both domains. Protective factors included higher education and alcohol consumption. These results suggest that targeting cardiovascular risk factors in interventions in mid-life may have a greater effect on future attention/executive function than memory function, whereas targeting hyperglycemia or diabetes could be beneficial for preserving multiple cognitive domains. In addition, effects of different risk factors may vary depending on the *APOE* genotype.

### Electronic supplementary material

Below is the link to the electronic supplementary material.

Supplementary Material 1

## Data Availability

Data was acquired from the MPP/MDCS Steering Committee and they have not given their permnission for researchers to share their data. Detailed instructions for data requests can be found on: https://www.malmo-kohorter.lu.se/malmo-cohorts.
