# Supplementary material for: Associations of modifiable and non-modifiable risk factors with cognitive functions – a prospective, population-based, 17 years follow-up study of 3,229 individuals
Source: Alzheimers Res Ther. 2024 Jun 26;16:135. doi: 10.1186/s13195-024-01497-6 (PMC11202373; doi:10.1186/s13195-024-01497-6)
Supplement: Supplementary file 1 — Supplementary Material 1 [file 13195_2024_1497_MOESM1_ESM.docx]

**Supplementary Material**

**Supplementary eTable 1. Non-significant predictors of future memory function.**

| Baseline Predictor | Univariate model^a^ | Basic model^b^ |
| --- | --- | --- |
|  | β (95% CI) | β (95% CI) |
| Carotid stenosis | -0.14 (-0.21 – -0.08)*** | -0.04 (-0.11 – 0.03) |
| Hypertension (yes/no) | -0.14 (-0.25– -0.04)** | -0.10 (-0.20 – 0.01) |
| LDL-C | -0.06 (-0.09 – -0.02)** | -0.01 (-0.05 –0.03) |
| Physical activity (METh/week) | -0.02 (-0.13 – 0.09) | 0.01 (-0.02 ­– 0.04) |
| Prevalent or incident stroke | -0.25 (-0.39 – -0.12)*** | -0.13 (-0.26 – 0.00) |
| Smoking (never smoker as reference)  Smoker  Former smoker | 0.04 (-0.05 – 0.13)  -0.06 (-0.14 – 0.02) | 0.00 (-0.09 – 0.10)  -0.04 (-0.13 – 0.04) |

^a^ Including only the predictor time between baseline and follow-up.

^b^ Including the predictor adjusted for age, sex, education, time between baseline and follow-up and prevalent or incident stroke.

* p<0.05, ** p<0.01, *** p<0.001

**Supplementary eTable 2. Non-significant predictors of future attention/executive function.**

| Baseline Predictor | Univariate model ^a^ | Basic model ^b^ |
| --- | --- | --- |
|  | β (95% CI) | β (95% CI) |
| *APOE-genotype*  ɛ3/ɛ3  ɛ2/ɛ2 or ɛ2/ ɛ3  ɛ2/ɛ4  ɛ3/ɛ4 or ɛ4/ɛ4 | Reference  -0.00 (-0.11 – 0.11)  -0.18 (-0.41 – 0.04)  -0.02 (-0.10 – 0.07) | Reference  0.02 (-0.09 – 0.13)  -0.11 (-0.34 – 0.12)  0.01 (-0.08 – 0.10) |
| Carotid stenosis | -0.10 (-0.16 – -0.03)** | -0.01 (-0.08 – 0.06) |
| LDL-C | -0.03 (-0.07 – 0.00) | 0.03 (-0.01 – 0.07) |
| Number of pack years (smoking) | -0.00 (-0.00 – 0.00) | 0.00 (-0.00 – 0.01) |
| Physical activity (METh/week) | -0.00 (-0.03 – 0.03) | -0.01 (-0.04 – 0.03) |
| Smoking  Never smoker  Smoker  Former smoker | Reference  -0.09 (-0.18 – 0.00)  -0.03 (-0.11 – 0.00) | Reference  -0.04 (-0.14 – -0.05)  -0.01 (-0.09 – 0.08) |
| Systolic blood pressure (z-scores) | 0.12 (0.08 – 0.15)*** | 0.03 (-0.01 – 0.07) |
| Triglycerides (z-scores) | 0.08 (0.03 – 0.12)*** | 0.02 (-0.02 – 0.06) |

^a^ Including only the predictor time between baseline and follow-up.

^b^ Including the predictor adjusted for age, sex, education, time between baseline and follow-up and prevalent or incident stroke.

* p<0.05, ** p<0.01, *** p<0.001

**Supplementary eTable 3. Interaction effects with *APOE*-genotype.**

| Baseline Predictor | Memory function | Attention/Executive function |
| --- | --- | --- |
|  | β (95% CI) ^a^ | β (95% CI) ^a^ |
| Alcohol consumption ^b^  Q1 (0-0.23 standard drinks/day)  Q2 (0.24-0.56 standard drinks/day)  ɛ2/ɛ2 or ɛ2/ ɛ3  ɛ2/ɛ4  ɛ3/ɛ4 or ɛ4/ɛ4    Q3 (0.57-1.01 standard drinks/day)  ɛ2/ɛ2 or ɛ2/ ɛ3  ɛ2/ɛ4  ɛ3/ɛ4 or ɛ4/ɛ4    Q4 (1.02-7.5 standard drinks/day)  ɛ2/ɛ2 or ɛ2/ ɛ3  ɛ2/ɛ4  ɛ3/ɛ4 or ɛ4/ɛ4 | Reference  0.28 (-0.05 – 0.61)  0.76 (0.08 – 1.43)  0.21 (-0.05 – 0.46)  0.39 (0.07 – 0.71)  0.37 (-0.23 – 0.98)  0.24 (-0.02 – 0.49)  0.28 (-0.04 – 0.61)  0.59 (-0.10 – 1.28)  0.18 (-0.06 – 0.43) | Reference  0.41 (0.09 – 0.74)*  0.33 (-0.34 – 0.99)  0.32 (0.07 – 0.58)*  0.38 (0.06 – 0.70)*  0.38 (-0.22 – 0.98)  0.35 (0.10 – 0.60)**  0.30 (-0.02 – 0.62)  0.06 (-0.22 – 0.98)  0.24 (0.00 – 0.49)* |
| BMI (z-scores)  ɛ2/ɛ2 or ɛ2/ ɛ3  ɛ2/ɛ4  ɛ3/ɛ4 or ɛ4/ɛ4 | 0.02 (-0.09 – 0.13)  0.01 (-0.20 – 0.22)  0.06 (-0.03 – 0.15) | 0.00 (-0.11 – 0.11)  0.01 (-0.20 – 0.22)  -0.09 (-0.18 – -0.01)* |
| Education  ≤8 years  9-12 years  ɛ2/ɛ2 or ɛ2/ ɛ3  ɛ2/ɛ4  ɛ3/ɛ4 or ɛ4/ɛ4  >12 years  ɛ2/ɛ2 or ɛ2/ ɛ3  ɛ2/ɛ4  ɛ3/ɛ4 or ɛ4/ɛ4 | Reference  -0.10 (-0.35 – 0.15)  0.39 (-0.15 – 0.24)  -0.06 (-0.26 – 0.14)  -0.07 (-0.37 – 0.24)  0.10 (-0.56 – 0.76)  -0.26 (-0.48 – -0.03)* | Reference  0.17 (-0.01 – 0.60)  0.17 (-0.36 – 0.70)  0.09 (-0.10 – 0.29)  0.30 (-0.01 – 0.60)  -0.06 (-0.72 – 0.59)  -0.10 (-0.33 – 0.13) |
| HbA_1c_ (z-scores)  ɛ2/ɛ2 or ɛ2/ ɛ3  ɛ2/ɛ4  ɛ3/ɛ4 or ɛ4/ɛ4 | -0.02 (-0.14 – 0.11)  0.26 (-0.03 – 0.55)  -0.05 (-0.13 – 0.04) | -0.29 (-0.51 ­– -0.08)**  -0.62 (-1.18 –-0.06)*  -0.01 (-0.15 – 0.13) |
| HDL-C (z-scores)  ɛ2/ɛ2 or ɛ2/ ɛ3  ɛ2/ɛ4  ɛ3/ɛ4 or ɛ4/ɛ4 | 0.05 (-0.07 – 0.17)  0.25 (0.01 – 0.48)*  0.04 (-0.05 – 0.13) | 0.05 (-0.06 – 0.17)  -0.09 (-0.32 – 0.15)  -0.02 (-0.11 – 0.07) |
| Sex (0=male)  ɛ2/ɛ2 or ɛ2/ ɛ3  ɛ2/ɛ4  ɛ3/ɛ4 or ɛ4/ɛ4 | 0.11 (-0.12 – 0.34)  0.42 (-0.06 – 0.91)  -0.03 (-0.21 – 0.14) | -0.07 (-0.30 – 0.15)  -0.26 (-0.74 – 0.22)  -0.05 (-0.22 – 0.13) |
| Stroke (prevalent & incident)  ɛ2/ɛ2 or ɛ2/ ɛ3  ɛ2/ɛ4  ɛ3/ɛ4 or ɛ4/ɛ4 | -0.05 (-0.43 – 0.34)  -0.79 (-1.61 – 0.04)  -0.37 (-0.69 – -0.05)* | -0.20 (-0.58 – 0.19)  -0.32 (-1.14 – 0.51)  -0.40 (-0.72 – -0.08)* |

See Supplementary Fig. 1-2 for visualization of interaction effects.

**^a^** Model including the predictor, APOE-genotype and the interaction effect between the two, adjusted for age, sex, education, time between baseline and follow-up and prevalent or incident stroke.

^b^ Alcohol consumption in quartiles with lowest quartile as reference: Q1 = 0-0.23 standard drinks/day (0-3.37g/day); Q2 = 0.24-0.56 standard drinks/day (3.38- 7.83g/day); Q3: 0.57-1.01 standard drinks/day (7.84-15.2g/day); Q4 = 1.02-7.5 standard drinks/day, (15.3-105g/day).

* p<0.05, ** p<0.01, *** p<0.001

**Supplementary eFigure 1. Significant interaction effects between predictors and *APOE*-genotype on memory function.**

**
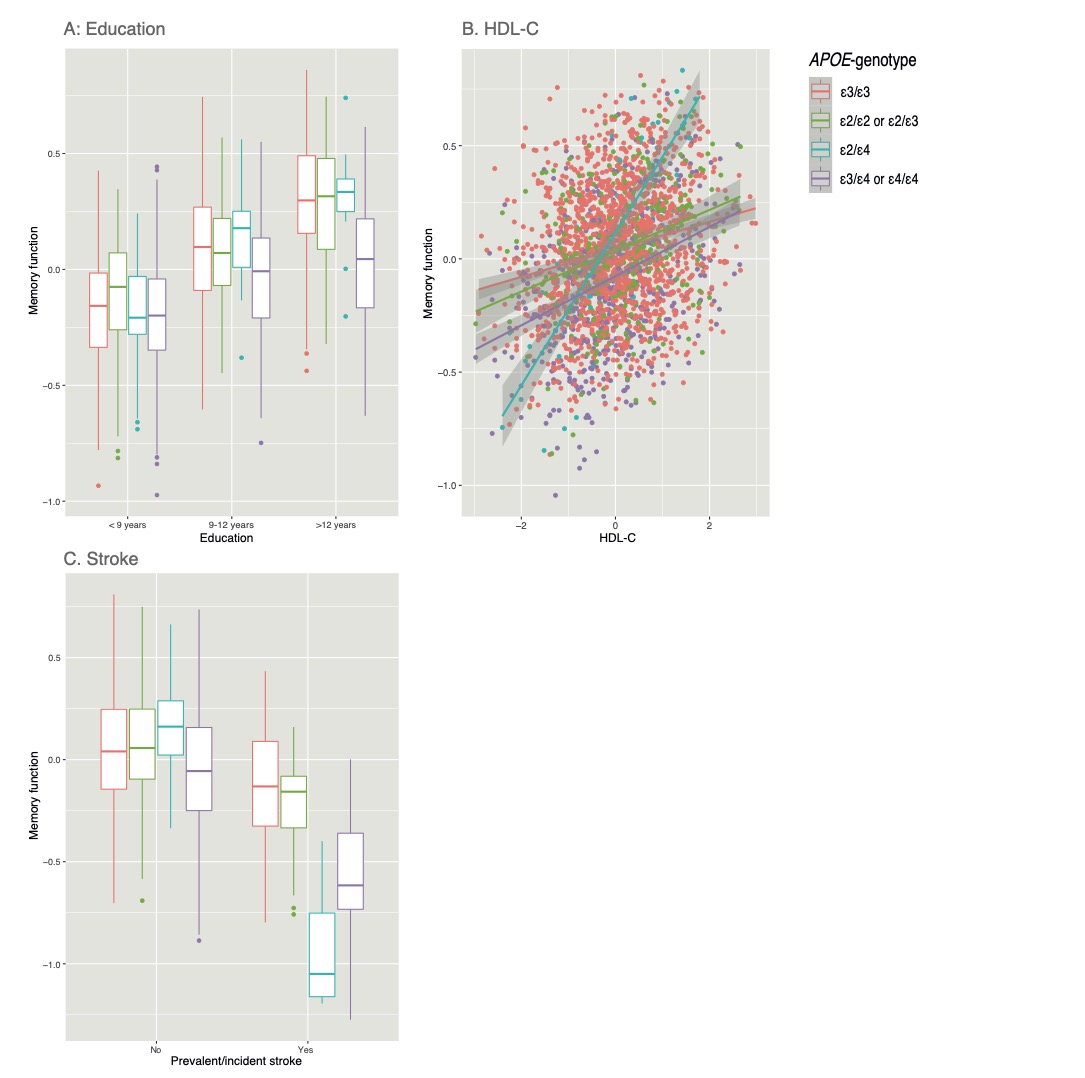
**

**Supplementary eFigure 2. Significant interaction effects between predictors and *APOE*-genotype on attention/executive function.**

**
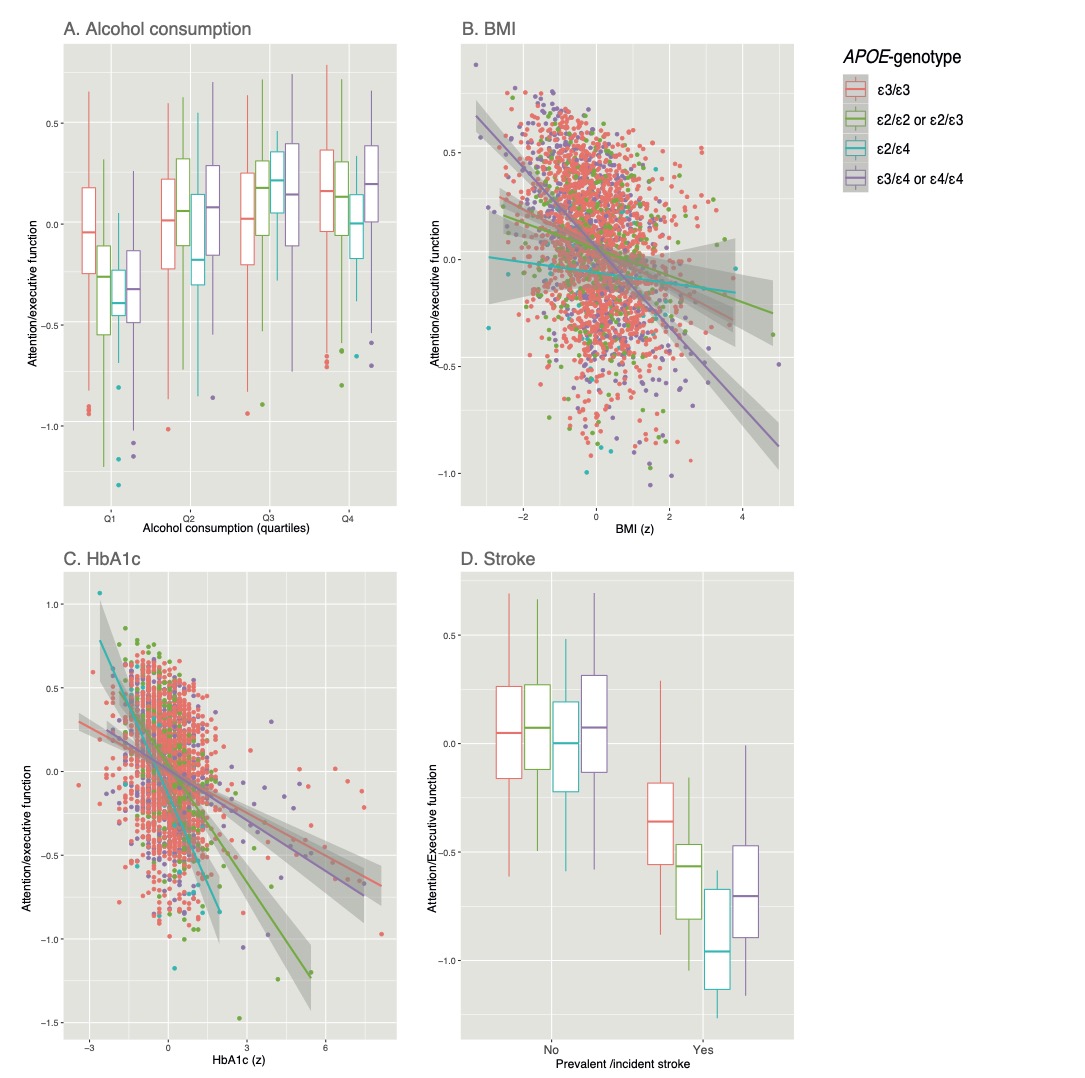
**

**Supplementary eTable 4. Alcohol consumption sensitivity analyses.**

| Baseline Predictor | Basic model ^a^ (Memory, MMSE) | Multivariable model ^b^ (Memory, MMSE) | Basic model ^a^ (Executive function, AQT) | Multivariable model ^c^ (Executive, AQT) |
| --- | --- | --- | --- | --- |
|  | β (95% CI) | β (95% CI) | β (95% CI) | β (95% CI) |
| Excluding participants with zero alcohol comsumption |  |  |  |  |
| Alcohol consumption ^d^  Q1 (≤0.23 standard drinks/day)  Q2 (0.24-0.56 standard drinks/day)    Q3 (0.57-1.01 standard drinks/day)    Q4 (≥1.02 standard drinks/day) | Reference  0.05 (-0.07 – 0.18)  0.10 (-0.03 – 0,22)  0.09 (-0.04 – 0.22) | Reference  0.07 (-0.06 – 0.20)  0.09 (-0.04 – 0.22)  0.10 (-0.04 –0.23) | Reference  0.15 (0.03 – 0.27)*  0.17 (0.05 – 0.30)**  0.25 (0.13 – 0.38)*** | Reference  0.13 (0.00 – 0.26)*  0.15 (0.02 – 0.28)*  0.22 (0.08 – 0.35)** |
| Lancet Commission(5) cutoff for suggested increased risk of dementia |  |  |  |  |
| Alcohol >1.7 standard drinks/day | -0.06 (-0.18 – 0.06) | -0.01 (-0.14 – 0.12) | 0.13 (0.01 – 0.25)* | 0.11 (-0.01 – 0.24) |
| Alcohol >1.7 standard drinks/day (excluding zero consumers) | -0.07 (-0.19 – 0.05) | -0.03 (-0.16 – 0.10) | 0.11 (-0.01 ­– 0.23) | 0.09 (-0.03 – 0.22) |

^a^ Model including the predictor adjusted for age, sex, education, time between baseline and follow-up and prevalent or incident stroke.

^b^ One model combining all significant predictors from the basic models (Table 2), adjusted for age, sex, education, time between baseline and follow-up, prevalent or incident stroke and blood lipid lowering medication.

^c^ One model combining all significant predictors from the basic models (Table 3), adjusted for age, sex, education, time between baseline and follow-up and blood lipid lowering medication.

^d^ Alcohol consumption in quartiles with lowest quartile as reference: Q1 ≤0.23 standard drinks/day (0-3.37g/day); Q2 = 0.24-0.56 standard drinks/day (3.38- 7.83g/day); Q3: 0.57-1.01 standard drinks/day (7.84-15.2g/day); Q4 ≥1.02 standard drinks/day, (≥15.3g/day).

* p<0.05, ** p<0.01, *** p<0.001

| Predictors ^a^ | Odds Ratios | 95 % CI | *p* |
| --- | --- | --- | --- |
| Age | 0.63 | 0.58 – 0.68 | **<0.001** |
| Alcohol consumption ^b^  Q1 0-0.23 standard drinks/day  Q2 (0.24-0.56 standard drinks /day)  Q3 (0.57-1.01 standard drinks/day)  Q4 (1.02-7.5 standard drinks/day) | Reference  1.74  1.92  1.82 | Reference  1.42 – 2.14  1.55 – 2.38  1.46 – 2.27 | **Reference**  **<0.001**  **<0.001**  **<0.001** |
| *APOE genotype*  ɛ3/ɛ3  ɛ2/ɛ2 or ɛ2/ ɛ3  ɛ2/ɛ4  ɛ3/ɛ4 or ɛ4/ɛ4 | Reference  1.13  0.66  0.88 | Reference  0.90 – 1.41  0.44 ­– 1.01  0.74 – 1.03 | Reference  0.298  0.052  0.118 |
| BMI | 0.97 | 0.90 – 1.04 | 0.372 |
| Cholesterol | 1.05 | 0.98 – 1.13 | 0.192 |
| Education  ≤8 years  9-12 years  >12 years | Reference  1.20  1.23 | Reference  1.02 – 1.41  1.01 – 1.50 | **Reference**  **0.027**  **0.039** |
| HbA_1c_ | 0.81 | 0.75 – 0.87 | **<0.001** |
| Hypertension | 0.74 | 0.64 – 0.86 | **<0.001** |
| Sex (0=male, 1=female) | 1.18 | 1.01 – 1.38 | **0.036** |
| Stroke | 0.62 | 0.51 – 0.77 | **<0.001** |

**Supplementary eTable 5. Predictors of attending the follow-up visit.**

This analysis was performed on n=6,103 participants including both the sample used in the present study (n=3,229) and those that did not attend the follow-up visit, but had data from the baseline visit (n=2,369).

^a^ All continuous variables as z-scores (i.e, not alcohol, *APOE*, education, hypertension, sex and stroke). One model combining all predictors.

^b^ Alcohol consumption in quartiles with lowest quartile as reference: Q1 = 0-0.23 standard drinks/day (0-3.37g/day); Q2 = 0.24-0.56 standard drinks/day (3.38- 7.83g/day); Q3: 0.57-1.01 standard drinks/day (7.84-15.2g/day); Q4 = 1.02-7.5 standard drinks/day, (15.3-105g/day).

REFERENCES

1. Livingston G, Huntley J, Sommerlad A, et al. Dementia prevention, intervention, and care: 2020 report of the Lancet Commission. Lancet 2020;396:413-446.
